# Prevalence and Characteristics of Abuse Experiences and Depression Symptoms among Injection Drug-Using Female Sex Workers in Mexico

**DOI:** 10.1155/2013/631479

**Published:** 2013-05-12

**Authors:** Monica D. Ulibarri, Sarah P. Hiller, Remedios Lozada, M. Gudelia Rangel, Jamila K. Stockman, Jay G. Silverman, Victoria D. Ojeda

**Affiliations:** ^1^Department of Psychiatry, University of California, San Diego, 9500 Gilman Drive, La Jolla, CA 92093-0849, USA; ^2^Division of Global Public Health, Department of Medicine, University of California, San Diego, 10111 N. Torrey Pines Road, La Jolla, CA 92093-0507, USA; ^3^Prevencasa, Avenida Baja California 7580, Zona Norte, 22000 Tijuana, BC, Mexico; ^4^El Colegio de La Frontera Norte-Secretaria de Salud, Carretera Escenica Tijuana-Ensenada, Km. 18.5, San Antonio del Mar, 22560 Tijuana, BC, Mexico

## Abstract

This mixed methods study examined the prevalence and characteristics of physical and sexual abuse and depression symptoms among 624 injection drug-using female sex workers (FSW-IDUs) in Tijuana and Ciudad Juarez, Mexico; a subset of 47 from Tijuana also underwent qualitative interviews. Linear regressions identified correlates of current depression symptoms. In the interviews, FSW-IDUs identified drug use as a method of coping with the trauma they experienced from abuse that occurred before and after age 18 and during the course of sex work. In a multivariate linear regression model, two factors—ever experiencing forced sex and forced sex in the context of sex work—were significantly associated with higher levels of depression symptoms. Our findings suggest the need for integrated mental health and drug abuse services for FSW-IDUs addressing history of trauma as well as for further research on violence revictimization in the context of sex work in Mexico.

## 1. Introduction

The United Nations defines violence against women as “any act of gender-based violence that results in, or is likely to result in, physical, sexual or mental harm or suffering to women, including threats of such acts, coercion or arbitrary deprivation of liberty, whether occurring in public or private life [1].” Examples of gender-based violence include but are not limited to intimate partner violence, sexual violence, sexual abuse of female children, sexual harassment and intimidation in the workplace, and commercial sexual exploitation [2, 3]. Various forms of gender-based violence against female sex workers (FSWs) have been documented worldwide [4–13]. Likewise, there is extensive literature examining the mental health consequences for women in general of gender-based violence [14–18]. However, less research has been done on the relationships between FSWs' mental health status and gender-based violence, especially among FSWs who inject drugs (FSW-IDUs) in Mexico. 

Several studies find that many FSWs are enmeshed in chronic patterns of violence victimization and substance use resulting in elevated levels of psychological distress [8, 9, 19–21]. Most of this research has focused on psychological distress rather than on depression symptoms. FSWs' early exposure to violence, such as childhood sexual abuse, may have long-term mental health consequences. For example, Surratt et al. [20] found that history of childhood abuse and recent violence victimization by clients were associated with serious mental illness among street-based FSWs in Miami, Florida. Cwikel et al. [22] found that childhood trauma, including childhood sexual abuse, was correlated with depression and posttraumatic stress disorder symptoms among a group of trafficked FSWs awaiting deportation from Israel. Additionally, drug abuse is often more prevalent among FSWs with a history of childhood abuse and violence victimization by clients [9, 10, 23]. This study will add to the knowledge of history of violence victimization and mental health among drug-using Mexican women by examining the context of first abuse experiences and the subsequent impact of those experiences on the prevalence of depression symptoms, substance use, and violence revictimization in the sex work context. 

To our knowledge, this is one of the first mixed methods studies to provide information regarding the prevalence and contexts of childhood abuse and of gender-based violence in adulthood among a large sample of FSW-IDUs along the Mexico-US border, a region where violence, drug use, and sex work often intersect [24]. In two of the largest cities of the region—Tijuana, Baja California, and Ciudad Juarez, Chihuahua—sex work is legal in specified zones of tolerance. These cities are also located along major drug-trafficking routes, which contribute to their having the highest rates of drug use and fastest growing populations of injection drug users in Mexico [24]. Information gained from this study may help elucidate the relationships between gender-based violence, substance use, and depression among FSW-IDUs. Moreover, these data may aid in improving interventions for this highly stigmatized and vulnerable population that frequently lacks access to adequate mental health services and substance abuse treatment [25].

The purpose of this study was to describe the prevalence and characteristics (e.g., types of abuse, perpetrators, disclosure, support received, etc.) of physical and sexual abuse experiences in childhood (before age 18) and adulthood (after age 18) and associations with current symptoms of depression among FSW-IDUs in Tijuana and Ciudad Juarez, Mexico. This mixed methods study analyzed quantitative data related to the prevalence and correlates of abuse. Qualitative interviews conducted in Tijuana enabled us to further contextualize the quantitative findings. Based on the Syndemic Model for Hispanics [26] and syndemic theory [19, 27], which posit that substance abuse, violence, and mental health issues such as depression interact to account for health disparities among marginalized groups, the current study hypothesized that among Mexican FSW-IDUs, there would be high rates of childhood abuse history and of client-perpetrated abuse and that history of abuse and substance use would be related to current depression symptoms. 

## 2. Method

### 2.1. Participants

This study utilized baseline interview data collected between October 2008 and July 2010 for a behavioral intervention study to reduce injection and sexual risk among FSW-IDUs in Tijuana and Ciudad Juarez, Mexico [28, 29]. A total of 624 FSW-IDU participated (Tijuana = 308; Ciudad Juarez = 316). Eligibility criteria were being of age 18 years or older; having had unprotected vaginal or anal sex with a male client in the previous month; having injected illicit drugs and shared syringes and/or other injection equipment within the past month; ability to speak Spanish or English; ability to provide informed consent; having no plans to move out of the city permanently in the following 18 months; and agreeing to undergo free STI treatment. More detailed information regarding the participants, recruitment, and the intervention study are available in a previous publication [29].

### 2.2. Procedure for Quantitative Interviews

 The study utilized a targeted sampling technique. Outreach workers recruited potential participants from venues known to be frequented by FSWs and IDUs which included bars, street corners, alleys, motels, hotels, brothels, and shooting galleries. Potential participants were approached about the study and those who were interested in participating in the study were referred to the project offices or a mobile unit for eligibility screening. Eligible women provided written informed consent. Participants underwent interviewer-administered surveys in English or Spanish at baseline and at 4-, 8-, and 12-month followups; however, only baseline data were used for this study. FSW-IDUs who completed the surveys were compensated $25 USD. The study protocol was reviewed and approved by the institutional review boards at UC-San Diego, Tijuana General Hospital, and the Universidad Autónoma de Ciudad Juarez.

### 2.3. Quantitative Measures

The depression symptoms measure for this study was selected for cultural appropriateness and validity with Spanish-speaking, low-income populations. The quantitative interview consisted of additional questions developed by the research team. Questions not already available in Spanish from previous studies with FSWs in Mexico [30, 31] were translated into Spanish and back-translated into English and reviewed by the study staff to make sure they retained their original meaning. Prior to implementation of the study, all quantitative items were reviewed for gender and cultural sensitivity by our bilingual, bicultural, and binational (U.S.-Mexico) team of researchers, and the questionnaire was piloted with Mexican FSW-IDUs in Tijuana for appropriate language and cultural sensitivity.

#### 2.3.1. Demographics

Participants were asked several questions regarding their sociodemographics such as age, marital status, place of birth, migration history, level of education, income, and number of dependents. 

#### 2.3.2. Sex Work

Sex work items included age at initiation of sex work, age at which regular sex work began, and number of clients in the past month.

#### 2.3.3. Abuse

Participants were asked (yes or no) if they had ever been forced or coerced to have nonconsensual sex against their will; physically abused; forced or coerced into having sex against their will with a client; or physically abused by a client. A “yes” answer to any of those questions prompted additional questions about context, including: age at which the first and most recent abuse events happened; perpetrator (husband, boyfriend, regular partner, male relative, female relative, client, stranger, or specified other); whether participants had ever disclosed the abuse to anyone (yes/no) and, if so, to whom (family member, friend, therapist, crisis hotline, doctor, support group, specified other), and the perceived level of support they received (“no support,” “some support,” “a lot of support”).

#### 2.3.4. Depression

Symptoms of depression (past week) were assessed using a Spanish-language, 10-item, short form of the Center for Epidemiologic Studies, Depression Scale (CES-D [32]) which has been shown to be reliable for use with Latino populations [33]. Cronbach's alpha for the CES-D items used in this study was 0.85. Response choices ranged from 0 = “rarely or none of the time (<1 day)” to 3 = “most or all of the time (5–7 days).” The depression symptoms total score was computed by summing the responses of all ten CES-D items. Women who were missing responses to one or more items of the CES-D did not get a total score (*n* = 2) as recommended by Grzywacz et al. and were not included in the analysis regarding depression symptoms. Total scores had a possible range of 0 to 30, with scores above 10 indicating clinical levels of depression [33]. 

#### 2.3.5. Drug Use

Drug use was assessed by asking participants whether (yes/no) they had ever used any of a specified list of drugs (e.g., marijuana, heroin, methamphetamine, cocaine) and, if so, the age at which they first used the drug and the mode of usage (e.g., injection, smoking). Since the eligibility criteria for the intervention study included having injected drugs in the past month, FSW-IDUs were also asked about the frequency with which they had injected drugs in the past month. Response choices were once a month or less, 2-3 days a month, once a week, 2-3 days a week, 4–6 days a week, once a day, or more than one time per day. For our analysis we recoded this variable as a binary, that is, “daily” versus “nondaily” injection drug use. 

### 2.4. Procedure for Qualitative Interviews

Between August 1 and October 31, 2009, a subsample of 47 participants from Tijuana were recruited into a pilot qualitative study focused on migration experiences as they relate to sex work and drug use behavior. Similar interviews were not conducted in Ciudad Juarez due to limited financial and personnel resources. Participants were recruited using a maximum variation sampling approach [34] based on the region in Mexico in which the women were born (North, Central, or Southern, or the State of Baja California; *n* = ~15 participants per region). Additional IRB approval for the qualitative substudy was obtained from UC-San Diego and the Tijuana General Hospital. All participants underwent additional voluntary and informed consent for participating in the qualitative interviews. 

Trained bilingual interviewers conducted in-depth interviews lasting 30 to 90 minutes in private rooms in the study's storefront site situated in the *Zona Roja* (i.e., red light district) of Tijuana. Interviews were digitally recorded. Participants were interviewed in English or Spanish, depending on their preference, and were reimbursed $20 USD for their time and transportation costs. Questions were designed to elicit migration, drug use, and sex work histories. Questions included the following: “what was your life like in (city or origin)?,” “why did you leave (city of origin)?,” and “can you tell me what happened the first time you (used drugs/injected drugs/traded sex)?.” Participants were also asked to describe their lives in Tijuana and their hopes and aspirations for the coming year. Although experiences of abuse or traumatic events were not the original focus of the qualitative interviews, these issues were prevalent (with minor variations) in participants' stories and thus constituted an emergent theme that achieved saturation [35].

### 2.5. Quantitative Data Analyses

First, we examined descriptive statistics and frequencies for the demographic, sex work, abuse, and drug use variables (see Table 1). Comparisons between FSW-IDUs with CES-D scores above the clinical cut point of 10 versus FSW-IDUs with scores below the cut point were conducted using Wilcoxon rank-sum tests or Chi-square tests for categorical data. However, to assess correlates of depression symptoms, subsequent univariate and multivariate linear regressions were conducted using the CES-D total score (versus a dichotomized score) in order to maximize effect size, power, and measurement reliability [36]. Univariate linear regressions were performed for each variable of interest to identify significant correlates of depression symptoms [37]. Finally, multivariate linear regression using generalized estimating equations (GEE) was performed to identify factors independently associated with depression symptoms. We assessed for multicollinearity by examining tolerance and the variance inflation factor (VIF) in regular regressions, and it did not appear to be a problem. However, we decided to take a conservative approach and use GEE because of its ability to analyze correlated data [38]. The final multivariate regression model was developed using a manual procedure where all the variables of interest that attained a significance level *P* < 0.10 in univariate analyses were considered for inclusion in the final model [37, 39, 40]. The likelihood ratio statistic was used to compare models, retaining variables that were significant at the *P* ≤ 0.05 level. 

### 2.6. Qualitative Data Analyses

We analyzed FSW-IDUs' qualitative interview data in order to contextualize quantitative findings pertaining to situations of abuse. Interviews were transcribed and then analyzed in the language of the interview in order to preserve the meanings and modes of expression of the study population, which included extensive code-switching (i.e., simultaneous use of English and Spanish) and drug-culture terminology. We developed a structured coding scheme using the interview protocol as a guide. Next, we added open codes, which incorporated emergent themes pertaining to abuse (emotional, physical, or sexual, or an unspecified traumatic event) at different stages of women's lives (childhood, adulthood). The research team, all of whom were bilingual, independently applied these codes to cross sections of six interviews in order to refine and create nuanced codes. The coding scheme was finalized, and codes were applied to all of the narratives using Atlas.ti software [41]. Analysis followed a general inductive approach according to which investigators focused on generating themes and identifying relationships between them. If new codes emerged, the coding scheme was updated and the transcripts were reread and coded according to the new structure. For the purpose of brevity and clarity, quotes are presented in English translation (performed by author SPH); Spanish language quotes are available upon request. All names are pseudonyms and do not reflect the participants' real identities. 

## 3. Results

 Participants' sociodemographic characteristics are displayed in Table 1. Of the 624 women who participated, 308 were from Tijuana and 316 from Ciudad Juarez. Almost all (93%) were born in Mexico. The average age was 33.7 years (SD: 8.4 years), and the mean highest year of school completed was 7.1 (SD = 3.0). In regards to marital status, 49% (*n* = 304) were single or never married. The majority reported having children (92%, *n* = 573). The mean number of children was 3.1 (SD = 1.7). The average age when participants first began regularly working as a sex worker was 21.3 years (SD = 6.8). By design, all of the women in the study were injection drug users; the majority (94%, *n* = 585) reported injecting drugs one or more times daily. The mean depression symptoms score for the sample was 17.5 (SD = 7.0), with 86% (*n* = 538) scoring above the suggested cut point (total score = 10) for depression [33]. 

### 3.1. Prevalence and Timing of Abuse

The quantitative data indicated that 50% (*n* = 313) of the women had ever experienced forced or coerced sex; 33% (*n* = 207) reported that the first forced sex event occurred before the age of 18 years. The mean age at which FSW-IDUs first experienced forced sex was 16.8 years (SD = 8.5). Furthermore, for 28% (*n* = 176), the first occurrence of forced sex preceded their entrance into sex work. In regards to physical abuse, 49% (*n* = 304) reported that they had ever been physically abused; 24% (*n* = 151) reported physical abuse before age 18. The mean age of first physical abuse for this sample was 19.9 years (SD = 8.3). 

### 3.2. Characteristics of First Childhood Abuse Experiences

Male family members (e.g., fathers, uncles) were the most common perpetrators of forced sex before age 18 (50%, *n* = 103), followed by strangers (27%, *n* = 57), as is shown in Table 2. The least commonly reported perpetrators were clients (4%, *n* = 8). In contrast to the forced sex data, husbands and boyfriends were the most common perpetrators of physical abuse before age 18 (55%, *n* = 83). Disclosure rates of first coerced sexual and physical abuse experiences that occurred before age 18 were 53% and 51%, respectively. Family members (especially mothers) were the most common people to whom the women disclosed their abuse experiences (78% for first sexual abuse and 75% for first physical abuse). For those who disclosed their sexual abuse, 47% (*n* = 51) reported receiving “no support.” Among those who disclosed the first time they were physically abused, 38% (*n* = 29) reported receiving “no support.” 

Participants' qualitative narratives revealed greater detail about perpetrators and about disclosures of forced sex that occurred in childhood. Reactions to women's disclosures of abuse varied. Some women reported confrontations with or even prosecution of perpetrators, while other women reported that their disclosures of abuse were met with disbelief or, in a few cases, blame for “seducing” the perpetrator. Several women linked childhood forced sex to current psychological symptoms, including substance abuse and emotional numbness. 

In the following quote, Alma, age 40, discusses how her mother refused to believe her when she disclosed that her stepfather had raped her. She also states that the sexual abuse and her mother's reaction to her disclosure of it influenced her drug use.

*The man who I thought was my father was not. When I was 8 he raped me, it was very ugly, that's why I tell you that I've never known true love, I think (crying)… yes, I've had partners, but I do not feel anything sentimentally… I've never felt it, with anyone… My stepfather when he penetrated me, when he raped me, after a year he left and did not come back… I was the only one who knew why… And then after, telling your mother and having her tell you that it is not true, that maybe it is not true, maybe I had flirted with him or something, well that's even worse, because a little girl, how can she flirt with an older man? I was barely an 8-year-old girl… Maybe that's why I started with the drugs—no, no, no, I won't make excuses, because let me tell you something, the drugs have their own allure, like, I did not start using because of the abuse, no, but [the rape] had something to do with it. *

Clara, age 38, explains how she turned to drugs to forget her mother's physical, emotional, and sexual abuse. She believes the drugs help her attenuate painful memories and make her mind “blank”:

*For me, [the drug] was a blessing, because I was going to start thinking about all the problems I had in my childhood, and for me, there was no better way to clear my mind, make it blank… because my mother hit me too much, in excess, she told me that she did not want me… when I was born, and many other things, including hurting me personally, on my body… how can I say, well, she put her hand in me, she molested me [sexually].*

### 3.3. Revictimization after Childhood Abuse

 Several participants reported leaving home to escape abuse as early as 8 or 9 years old, sometimes through a romantic relationship. Often, these participants left home and entered into risky environments (e.g., living on the street, engaging in sex work); some were revictimized by clients or partners. Elisa's narrative stands out because it combines many of these elements. Elisa, aged 41, left her home in a city on the U.S.-Mexico border at the age of 10 dressed as a boy and rode trains to Mexico City to escape her father's abuse, which consisted of beatings and being chained inside the house. In Mexico City, she lived on the street for almost a year before she was found; her father took her back to their hometown, where the abuse continued. When she was 12, her brother nearly raped her, and her mother refused to help. Elisa left with an older man who beat her and forcibly injected her with heroin.

*When I returned to [border town], I got together with this boy, well, he was a man, 18 years older than me, because my father kept hitting me, and took me back to the house and chained me up again. I told him to loosen the chain because it was too tight, [but really it was] so that I could get myself loose, because my brother wanted to rape me, one Christmas he wanted to rape me, he had me with a knife in my back and he told me that if I screamed he would kill me, but I got my courage together and screamed, my father came and beat him with the chain [that I was tied up with] but did not release me. I continued to be tied up, so I told my mom to tell my father to let me go, and no, she did not tell him, she stood me up and tied me around the waist, I was tied up like that for a week… so then I told him that I was going to leave, and he asked me where I would go, I told him I would go far away so he wouldn't be watching me, or that if he let me go I would get together with the first man who passed by, and he did not believe me. And a man passed there and I went with him, and it was with him that I had 4 children… but you have to realize, it was like going from bad to worse, because this guy hit me too. *

Another participant, Anayeli, age 32, reported leaving home at age 10 with her sister and a female neighbor to escape her brother's sexual abuse, but she soon discovered that her neighbor was a sex trafficker, and she believes that her mother was complicit in the trafficking. At age 11 she escaped sex work by finding a boyfriend, who also abused her.

*I did not want to be in my house because of what had happened [being sexually abused and trafficked] and everything turned out worse. The good thing was I found this boy, and he did help me, but he drank a lot and he beat me… the scars I have aren't from [injecting] drugs as much as the beatings, burns from him, we went through these cycles [of violence], and I withstood it until I couldn't anymore and left. *

### 3.4. Client-Perpetrated Abuse

Quantitative data indicated that 20% (*n* = 123) of the participants had ever experienced client-perpetrated physical abuse and 22% (*n* = 139) had ever experienced forced sex by clients. In the qualitative interviews, several FSW-IDUs described instances of client attacks, some of which were spontaneous or drug-fueled outbursts, while others seemed premeditated, where the client clearly intended to harm or even kill the participant. 

Sara, age 23, relates how she has gained a “gut feeling” about potentially dangerous clients and described an example of a recent attack. A client first paid for a sex act, but his use of methamphetamine affected his ability to attain an erection, so he demanded that Sara return his money. When Sara tried to leave, the client attacked and robbed her and left her unconscious on the sidewalk. The question we asked Sara was “is it dangerous to work in this, selling sex here in Tijuana?” Her response was as follows:

*Yes, because there are men that, well, with time you get to know when one is going to try to pull something on you, at first no, but now I can detect if a man is really handsome and has a fancy car and offers me a lot of money, but if I feel that my heart tells me not to go, I do not go, even if he gives me [a lot], I do not go, because I know he is not good, you understand?… One time, this guy wanted to strangle me, he choked me, we went and I had already gone out with him once before, but he used meth, and all of a sudden he couldn't get it up, and I do not know what happened, and he had already paid me $40, which I threw in my bag and I was going to get out because we were driving in his car. Then he grabs me and starts choking me, and when he finished, well, I lost consciousness, when I woke up I was on the sidewalk, he had taken $20 and left me $20, I called my boyfriend and he came and got me. The next day, the bruises were all the way up to my eyes, I couldn't move my neck.*

Other women reported that they began to use injection drugs to help forget painful memories of having been attacked by clients. Liz, age 45, describes being taken by a client to a vacant lot, where he beat and raped her:

*One thing happened with this guy, he took me to a vacant lot, and he really fucked me up, he almost killed me, and that traumatized me a little, more because the guy, I couldn't do anything to him at that moment, and I did not know what to do, the guy was still in [town] and he kept harassing me, so this event with this guy left me traumatized, and then I found out about heroin, and with heroin, I forgot everything, and I said to myself, “this is what I need.”*

### 3.5. Correlates of Depression Symptoms

 We examined correlates of depression symptoms in univariate linear regressions in the full sample as shown in Table 3. Level of education was the only demographic variable associated with depression symptoms (*β* = −0.21, *P* = .03, 95% CI [−0.41, −0.02]). FSW-IDUs with higher levels of depression symptoms were more likely to have ever experienced forced sex (*β* = 2.32, *P* < .001, 95% CI [1.23,3.41]), ever been physically abused (*β* = 1.66, *P* < .001, 95% CI [0.55,2.76]), ever been physically abused by a client (*β* = 1.90, *P* = .01, 95% CI [0.55,3.25]), and ever experienced forced sex in the context of sex work (*β* = 2.65, *P* < .001, 95% CI [1.39,3.90]). None of the drug abuse variables were significantly associated with depression symptoms.

In the final multivariate linear regression model (see Table 4), two factors remained independently associated with depression symptoms. FSW-IDUs who reported ever experiencing forced sex were more likely to experience current depression symptoms (*β* = 1.49, *P* = .02, 95% CI [0.22,2.76]) as were FSW-IDUs who experienced forced sex in the context of sex work (*β* = 1.76, *P* = .03, 95% CI [0.21,3.32]).

## 4. Discussion

 The purpose of this study was to examine the prevalence and characteristics of abuse experiences as well as factors associated with depressive symptoms among FSW-IDUs in Tijuana and Ciudad Juarez, Mexico. Quantitative data regarding FSW-IDUs' abuse experiences were contextualized by qualitative interviews. As hypothesized, there was a high prevalence of history of childhood abuse and client-perpetrated violence victimization among the FSW-IDUs in this study, which is consistent with previous research with drug-using FSWs [20] and FSWs in Mexico [9]. Significantly, over 80% of the women in the study scored above the CES-D cut point for depression. This rate was very high compared to the general population of Mexican women [42] but consistent with other studies of street-based and drug-using sex workers worldwide [20, 22, 43]. It is important to note, however, that these studies did not use the same measures of depression or depression symptoms. For example, the Belló et al. [42] study used the DSM-IV criteria which is a more conservative estimate. Therefore, differences in depression rates may be attributed to measurement differences. In multivariate analyses, we found that ever having been raped and rape in the context of sex work were independently associated with depression symptoms, suggesting that the trauma of sexual violence may have adverse effects on FSW-IDUs' mental health. Although drug use was not significantly associated with depression symptoms in the quantitative results, women did mention using drugs as a coping mechanism to deal with the trauma of gender-based violence. 

 In-depth interviews with a subset of women residing in Tijuana provided context and additional information about FSW-IDUs' abuse experiences and reinforced quantitative descriptive findings regarding perpetrators and disclosure of abuse. Notably, FSW-IDUs commonly reported revictimization and the utilization of drugs to cope with psychological distress: several women reported leaving home as children to escape abuse, entering sex work or new relationships where they again experienced violence victimization and using drugs to numb the feelings and memories associated with the abuse. This is consistent with previous research in which childhood sexual abuse was found to be significantly associated with women's engaging in sex work [44, 45]. A strong relationship between victimization in childhood and victimization in adulthood has been identified previously [46, 47]. A recent study of abuse experiences among a national sample of 2,000 Latina women found that 54% reported at least one victimization experience during their lifetime, and of those, two-thirds (66%) experienced more than one victimization incident, which indicates high rates of polyvictimization and revictimization [48]. Revictimized women are more prone to adverse mental health outcomes (e.g., PTSD, depression) and substance abuse [49]. 

 Many of the women reported never disclosing their experiences of abuse, and those who did disclose experienced mixed results: some felt supported, whereas others were met with disbelief and victim blaming. Fear of negative responses to their disclosures likely accounted for the low disclosure rates in this sample, which are consistent with other studies' accounts of disclosure of childhood sexual abuse among women in Mexico [50] and Latina women of Mexican descent in the USA [51, 52]. Previous research in the USA has shown that women born in Mexico are less likely to report gender-based violence (such as intimate partner violence) than are Latina women born in the USA. This is likely due to many factors including immigration status, fear of interaction with police and other authorities, fear of retribution from their abuser, and stronger adherence to traditional Mexican cultural values, gender role orientation, and embarrassment [53, 54]. The Mexican cultural values of *familismo* (strong identification with and loyalty to the extended family with emphasis on respect, cooperation, and protection of family honor) [55] and *simpatía* (a deferential posture toward family members and others in efforts to maintain harmony) may hinder disclosure by victims wanting to preserve family unity or protect the perpetrator, especially if the perpetrator is a family member [51, 54, 56]. In a study of Latina women who had experienced intimate partner violence, Montalvo-Liendo et al. [54] found that many abuse victims did not want to worry their parents or “add to their problems” (pg. 364) by disclosing their abuse. At the same time, when Latina women do disclose their abuse, they do so most often to family members, perhaps because they anticipate receiving support due to *familismo* [51]. However, as is documented in this study and in one by Belknap and Cruz [50], women may be revictimized and experience additional trauma if they reveal abuse and are not believed or are even blamed for the abuse or physically punished. This area needs further research and should be taken into consideration when working with Latina populations. 

## 5. Limitations

Our findings are subject to several limitations. Most important, we did not conduct in-depth interviews in Ciudad Juarez due to limited resources. However, the quantitative analyses employed data from two major cities on the U.S.-Mexico border, thus maximizing the sample size and increasing power to detect significant effects. Second, the experiences that FSW-IDUs in Tijuana reported in qualitative interviews may not be generalizable to the experiences of the FSW-IDUs in Ciudad Juarez. Additional qualitative research is needed to understand the similarities or differences in abuse contexts among FSW-IDUs residing in Ciudad Juarez. However, we controlled for study site in our quantitative analysis to allow for possible site differences. Third, the participants in this study constituted a convenience sample and thus may not be representative of all FSW-IDUs in these cities or elsewhere. Fourth, the English to Spanish translation of some study questions does not guarantee their adequateness for this population. However, our U.S.-Mexico bicultural, bilingual study team reviewed all questionnaire items for gender and cultural sensitivity and piloted the questionnaire with Mexican FSW-IDU's prior to implementation. 

As with most violence research, another limitation of this study is that it was cross-sectional and thus of limited value in establishing causal relationships. Longitudinal studies that assess onset of abuse and correlates such as depression and drug use could further clarify our study findings. Finally, as mental health issues were not the focus of the qualitative interviews, women were not questioned specifically about them. Nevertheless, women's narratives, particularly around drug use as a coping mechanism, implied that their mental well-being had been adversely affected by their lifetime experiences of abuse. These remain topics for further study with this and other samples of FSW-IDUs who have experienced gender-based violence. 

## 6. Conclusions 

 This study examined details of abuse in greater depth than is common in most research on history of abuse among FSW-IDUs. The utilization of qualitative as well as quantitative data allowed us to more thoroughly explore contexts of abuse, which led to better understanding of women's experiences. Notably, analysis of both types of data led to similar conclusions. Our study suggests a cycle of violence victimization, in which women leave home early to escape abuse, only to enter sex work and be further at risk for violence victimization by clients. FSW-IDUs reported using drugs to cope with the trauma of childhood and adult abuse experiences. Using drugs, especially in the context of sex work, may increase the potential for revictimization, as drug-using FSWs may be more likely to encounter drug-related crime and violence and less likely to exercise clear judgment or be able to protect themselves. 

 This increased understanding of abuse experiences is necessary to inform future substance use and mental health treatment for this population. The findings suggest that FSW-IDUs in the Mexico-U.S. border region are in need of screening for history of abuse, more comprehensive and integrated care for mental health, substance use, and psychological trauma, and prevention of revictimization. However, this may be a challenge given the poor availability of mental health and substance use services available in Mexico [57]. As is the case for the general Mexican population, increased interventions for FSW-IDUs will require new government investment in the mental health services infrastructure; clinics and clinicians taking the initiative to screen for mental health, trauma, and substance use issues; and at the population level, increased awareness of mental health and substance use disorders and their treatments [57]. 

## Figures and Tables

**Table 1:** Background characteristics by depression symptom score.

Characteristic	Below depression Cut point (*n* = 84)	Above depression Cut point (*n* = 538)	Total (*N* = 624 )	*P*
Mean age	34.3 (SD = 8.7)	33.6 (SD = 8.6)	33.7 (SD = 8.4)	.75
Mean years of school completed	7.2 (SD = 3.2)	7.1 (SD = 2.9)	7.1 (SD = 3.0)	.12
Has children	77 (92%)	494 (92%)	573 (92%)	.96
Marital status				.39
Single/never married	35 (42%)	269 (50%)	304 (49%)	
Married/common law	40 (48%)	195 (36%)	237 (38%)	
Divorced	2 (2%)	14 (3%)	16 (3%)	
Separated	5 (6%)	40 (7%)	45 (7%)	
Widowed	2 (2%)	20 (4%)	22 (3%)	
Country of birth				.19
Mexico	81 (96%)	495 (92%)	578 (93%)	
USA	2 (2%)	40 (7%)	42 (7%)	
Other	1 (1%)	3 (1%)	4 (1%)	
Has lived in Tijuana/Ciudad Juarez whole life	36 (43%)	234 (44%)	270 (43%)	.91
Sex work variables				
Mean age when first engaged in sex work	20.5 (SD = 6.3)	20.1 (SD = 6.6)	20.2 (SD = 6.5)	.80
Mean age when began working regularly as sex worker	21.3 (SD = 6.9)	21.2 (SD = 6.8)	21.3 (SD = 6.8)	.81
Mean number of clients (past month)	50.9 (SD = 60.3)	49.1 (SD = 52.7)	49.3 (SD = 53.7)	.11
Abuse items				
Ever raped/forced sex	30 (36%)	283 (53%)	313 (50%)	**<.01**
Ever physically abused	35 (42%)	269 (50%)	304 (49%)	.19
Ever physically abused by client	15 (18%)	108 (20%)	123 (20%)	.61
Ever raped/forced sex during sex work	9 (11%)	130 (24%)	139 (22%)	**<.01**
Drug use variables				
Mean age when first injected drugs	22.8 (SD = 9.3)	21.6 (SD = 7.3)	21.8 (SD = 7.6)	**<.01**
Daily injector	76 (90%)	508 (94%)	585 (94%)	.16

Data are number (%) of women, unless, otherwise, indicated. Some frequencies do not sum to *n* due to missing values. Significant findings are in boldface.

**Table 2:** Characteristics of first childhood abuse experiences.

	First forced sex ≤ age 18 (*n* = 207)	First physical abuse ≤ age 18 (*n* = 151)
Perpetrator		
Husband/boyfriend/regular partner	17 (8%)	83 (55%)
Male relative	103 (50%)	35 (23%)
Client	8 (4%)	7 (5%)
Stranger	57 (27%)	17 (11%)
Other^a^	23 (11%)	9 (6%)
Disclosure		
Yes	110 (53%)	77 (51%)
No	95 (46%)	73 (49%)
Who they told		
Professional^b^	13 (12%)	7 (9%)
Family member	85 (78%)	58 (75%)
Friend	17 (16%)	16 (21%)
Other^c^	11 (10%)	9 (12%)
Amount of support received		
No support	51 (47%)	29 (38%)
Some support	29 (27%)	22 (29%)
A lot of support	29 (27%)	25 (33%)

Sums may not add to 100% because participants could indicate all that applied, missing data, or rounding.

^a^Specified others for perpetrators were family friends, neighbors, and acquaintances. ^b^Professionals included crisis hotline, counselor, social worker, doctor or nurse, and support group. ^c^Specified others to whom they disclosed were police, neighbor, and teacher.

**Table 3:** Univariate analysis.

	*β*	SE	95% CI	*P*
Demographics				
Age	−0.02	0.03	[−0.08,0.05]	.54
Marital status (married versus not married)	−0.45	0.59	[−1.62,0.71]	.45
Education	−0.21	0.10	[−0.41, −0.02]	**.03**
Number of children	−0.03	0.17	[−0.37,0.23]	.85
Site (interview location)	0.02	0.56	[−1.09,1.12]	.98
Lived in city of interview whole life	−0.73	0.57	[−1.84,0.38]	.20
Sex work variables				
Age when first began sex work	−0.02	0.04	[−0.10,0.07]	.73
Age when began working regularly as sex worker	−0.01	0.04	[−0.09,0.07]	.84
No. of clients in past month	0.01	0.01	[0.00, 0.02]	.24
Abuse items				
Ever raped/forced sex	2.32	0.56	[1.23,3.41]	**.00**
Ever physically abused	1.66	0.56	[0.55,2.76]	**.00**
Ever physically abused by client	1.90	0.69	[0.55,3.25]	**.01**
Ever raped/forced sex during sex work	2.65	0.64	[1.39,3.90]	**.00**
Drug use variables				
Age when first injected drugs	−0.03	0.04	[−0.10,0.05]	.49
Daily versus nondaily injectors	0.47	1.14	[−1.76,2.70]	.68
Ever used marijuana	−0.35	0.66	[−1.64,0.95]	.60
Ever used heroin	0.93	1.86	[−2.73,4.58]	.62
Ever used crack	−0.02	0.57	[−1.14,1.09]	.97
Ever used cocaine	−0.65	0.60	[−1.82,0.52]	.28
Ever used methamphetamine	−0.29	0.56	[−1.40,0.81]	.61
Ever used inhalants	0.75	0.64	[−0.51,2.00]	.24

CI: confidence interval. Significant findings are in boldface.

**Table 4:** Factors independently associated with depression symptoms.

	*β*	SE	95% CI	*P*
Education	−0.17	0.10	[−0.36, 0.02]	.08
Ever raped/forced sex	1.49	0.65	[0.22, 2.76]	**.02**
Ever physically abused	0.64	0.64	[−0.62, 1.90]	.32
Ever physically abused by client	0.12	0.85	[−1.55, 1.78]	.89
Ever raped/forced sex during sex work	1.76	0.79	[0.21, 3.32]	**.03**

CI: confidence interval. Significant findings are in boldface.
